# A Case Report Exploring Activity Intensity in Inpatient Rehabilitation after Stroke

**DOI:** 10.1155/2010/507476

**Published:** 2010-07-14

**Authors:** Kathryn Zalewski, Julie Kerk, Kristina Laundre, Amber Wacek, Melissa Wiedmeyer

**Affiliations:** ^1^College of Health Sciences, Department of Human Movement Science, Doctor of Physical Therapy Program, University of Wisconsin-Milwaukee, P.O. Box 413 Pavilion 360, Milwaukee, WI 53201, USA; ^2^Neurorehabilitation Unit, Department of Physical Therapy, Froedtert Memorial Lutheran Hospital, 9200 West Wisconsin Avenue, Milwaukee, WI 53226, USA

## Abstract

*Background and Purpose*. Inpatient rehabilitation in countries other than the United States (US) has been described as a time where patients are often not engaged in intensive physical activity. The purpose of this case report is to explore the amount and intensity of physical activity provided in inpatient rehabilitation after stroke in the US. *Methods*. This study presents a case report of a person admitted to an inpatient rehabilitation unit after sustaining a first stroke. A customized data collection tracked type of activity, activity intensity and social interaction every 5 minutes during the rehabilitation day. *Results*. 74 percent of the day was spent in low intensity, often seated, physical activity; 14% of the day was spent resting or sleeping. Only 2.91% the day was spent in moderate or high intensity activity with a mobility focus. *Conclusions*. Consistent with other studies, this case report suggests a relatively low physical demand to rehabilitation delivered in inpatient stroke rehabilitation. This case begins to raise questions about optimized rehabilitation parameters for acute stroke rehabilitation.

## 1. Introduction

Amount and duration of rehabilitation therapy delivered after stroke is largely influenced by regulations guiding reimbursement from federal funding programs. Inpatient rehabilitation is viewed as being beneficial to people in the early stages of stroke recovery [1]. The combined impact of physical therapy (PT), occupational therapy (OT), and speech therapy (ST) for individuals recovering from first stroke (CVA) is estimated at 20 points on the Functional Independence Measure (FIM) [2]. The functional impact of therapy is in part a function of dosage calculated by exploring daily physical activity (physical therapy sessions or nontherapeutic physical activity), total length of stay, and intensity of the sessions. Functional recovery, including the recovery of ambulation is tied to the total time spent practicing walking, and in particular time spend in high-intensity repetitive and task-specific practice [3]. Total activity dosage in inpatient rehabilitation, including therapeutic activity delivered in PT and nontherapeutic ambulation is not well described. 

On average, rehabilitation units in the United States provide 1.5–1.6 thirty minute sessions of physical, occupational and speech therapy daily [2, 4]. Physical therapy sessions are described to last 38 minutes. A majority of the sessions (81.1%) include some type of pregait or gait activities [4]. These observations suggest that most patients receive approximately 60 minutes of structured physical therapy daily. Nonstructured physical activity is harder to quantify; there are no known descriptions of the nontherapeutic activity in inpatient rehabilitation. 

An emerging body of evidence from stroke centers in other countries characterizes the acute rehabilitation window as lacking in activity intensity. A small study comparing the time use of people recovering from stroke in Belgium and Switzerland found discrepancies between hours spent in therapeutic activities (2.5 versus 4 hours), with PT the predominant therapy provided by both countries. In both countries, the bulk of the care was provided in the patient's room. The intensity of the PT services was not documented [5]. Bernhardt et al. documented stroke rehabilitation intensity in a hospital in Melbourne, Australia. Their findings suggest that people in the early stages of stroke recovery engage in moderate or high intensity activities less than 13 percent of the therapeutic day. Over 50% of the time, people in rehabilitation were resting in bed. This pattern of inactivity seemed to be independent of the frailty of the individual, as when the data were analyzed to remove those who were restricted to bed rest, the data were not significantly changed. In addition, people engaged in stroke rehabilitation were alone over 60% of the time. The authors conclude the inpatient rehabilitation stay is characterized by people being “inactive and alone” [6]. The finding of relative inactivity and social isolation was replicated in a case series exploring subacute rehabilitation [7]. While there is no known description of the comprehensive inpatient rehabilitation stay in the United States that examines both services delivered and intensity of those services delivered, if the rehabilitation environment parallels that of other countries, it is possible that the rehabilitation day is characterized by underactivity. 

The prospective payment system (PPS) used by the Centers for Medicare and Medicaid to reimburse care in inpatient rehabilitation facilities (IRFs) was passed as part of the Balance Budget Act in 1997 and implemented in 2002 [8]. While the full impact of the restructuring of rehabilitation payment is not known, trends towards shorter lengths of stay seem to be consistent [9], with lengths of stay for people recovering from stroke approximately 15 days (±12) [2]. O'Brien's recent review of trends in rehabilitation outcomes based on FIM score, discharge destination, and lengths of stay before and after introduction of PPS begin to suggest that while functional outcomes may not be changed with shorter lengths of stay, fewer individuals may be returning home [9]. A shorter length of stay, coupled with a rehabilitation environment characterized by underactivity creates the possibility that physical activity dosage in acute rehabilitation may be inadequate to meet the functional challenges of the patient and his/her family in the transition to a home environment. 

Undertreatment may have significant long-lasting implications for the stroke survivor. Recovery of independent ambulation is a critical component of stroke rehabilitation, and those who recover ability to ambulate live longer [10]. Recovery of ambulation after stroke appears to be associated with initial time to lower extremity weight bearing activity [11], and training in ambulation seems to translate to increased independence in other functional skills such as toilet transfers [12]. More minutes per day spent in PT gait activities were associated with higher discharge FIM scores and increased rates of discharge to home [12]. Predictors of stepping activity after stroke seem to be associated with amount and intensity of the walking training [13]. There are two concerns in rehabilitation associated with determining dosage associated with activity based interventions: (1) the amount of practice/repetition needed to build skills necessary for independence, and (2) the intensity of the practice/repetition needed to improve the cardiovascular conditioning necessary to meet the physical demands of daily life tasks as well as improving fitness and reducing risks associated with disuse/deconditioning. It is unclear whether the inpatient rehabilitation experience accomplishes either of these goals. 

If inpatient physical activity dosage with an emphasis on walking is critical to recovery of ambulation then an observation of the stroke rehabilitation environment may assist the rehabilitation team in optimizing an environment that will maximize outcomes. The purpose of this case report is to describe the physical demands of a person recovering from stroke in the early days of stroke recovery, admitted to an inpatient rehabilitation unit in the United States within 2 weeks of experiencing onset of first stroke. As a second objective the social interaction of the stroke rehabilitation is also reported. 

## 2. Case Description

This case report was developed to test methods for a larger study designed to explore the relationship between physical activity intensity in inpatient stroke rehabilitation and mobility outcomes. To improve sensitivity of the testing materials, the person selected for the case had to be completing the first week of inpatient rehabilitation and meet the criteria of: (1) medical stability meaning not confined to bed rest or restricted mobility for medical reasons, (2) able to participate in some mobility training both seated (e.g., wheelchair propulsion) and upright (gait or pregait activities), (3) require speech therapy services, and (4) have family involved in discharge planning. A physical therapist collaborator identified the first individual meeting this criteria after methods had been developed to participate in this case report. 

Data collection occurred in an inpatient stroke rehabilitation facility in a local teaching hospital representing the only stroke rehabilitation center accredited by the American Stroke Association in the geographic area. 

### 2.1. Patient

At the time of the data collection, EA was a 67-year-old married man admitted to a local inpatient rehabilitation center/Stroke Unit four days prior to participating in the data collection. After the study was described, he gave his assent to participate. EA was diagnosed with a first ischemic right middle cerebral artery infarct 12 days prior to admission and 16 days prior to data collection. At the time of data collection, according to the initial examinations of the occupational therapist and physical therapist assigned to EA, he required minimal assistance and set up with self-cares if completed in a seated position. He required moderate assistance with all upright mobility including pregait activities and supervision with seated wheelchair mobility over short distances. He had active use of the affected lower extremity (LE) and some voluntary activity of the affected upper extremity (UE), but did not spontaneously use the affected extremity for self-care including brushing teeth and shaving. He was right hand dominant. He was described as having normal receptive language skills, but slurred speech and mild short-term memory limitations by the speech pathologist. 

Discharge goals for EA were to return home with support, able to complete seated self-care skills with set up and requiring minimal assistance for upright tasks including transfers. He was expected to require minimal assistance for household level ambulation using an assistive device and to be independent with wheelchair mobility over even and uneven surfaces for short distances. Therapists anticipated his discharge to be 2 weeks from the date of data collection. 

### 2.2. Methods

A modification of methods used by Bernhardt et al. [6] was used to design the survey tool used for data collection. The original Bernhardt tool was presented to a small focus group consisting of 13 therapists: 9 physical therapists, 2 occupational therapists, and 2 speech therapists from the Stroke Unit. The tool was assessed by the focus group to determine whether any activities were missing, whether there was agreement on the intensity indicators, and for understanding of data collection methods. Revisions to the Bernhardt tool were based on the focus group feedback, and presented a second time to the focus group for consensus. The final version of the data collection tool used in this study is provided as an appendix. 

Four evaluators recorded EA's activity intensity and social contacts from 7:30 AM until 5:00 PM on a weekday in the first week of inpatient rehabilitation. Observations were collected every ten minutes between 7:30–9:00 and every 5 minutes from 9:00 AM until 5:00 PM to increase sensitivity of the observations. Observations between 9:00 and 10:00 were completed by all four raters to assess interrater reliability (percent agreement) of the tool; otherwise all observations were by a single rater. At each observation the activity category, intensity of the activity, and presence of others was noted on the scoring form according to the template presented in the appendix. The raters did not interact directly with EA. For observations of EA in the room, the observer witnessed the activity from outside the room. For observations in the unit, observations were made from a distance that did not allow for physical or verbal interaction with EA. 

The observation was recorded as unavailable if EA was off the unit, or if any person providing care or socializing with EA requested privacy. The highest level of activity witnessed at the observation moment (as opposed to the highest level of activity in the 5 minute observation window) was recorded. In total 103 observations were recorded; EA was off the unit for 3 observations resulting in 100 observations with data.

### 2.3. Results

Interrater reliability was evaluated using percent agreement; raters agreed 89% of the time on behavioral ratings indicating good reliability of the recording tool. Activity data are reported in Table 1.

Most (73.79%) of EA's day is spent in low-intensity activity typically involving sitting and engaging in self-care activities, cognitive rehabilitation, or upper extremity activities. Only 2.91% of EA's day is spent in activity that may present a significant aerobic challenge with a focus on mobility training. EA spent relatively little time resting in bed but not sleeping, but spent a large portion of the day sitting and resting. This may suggest that there is down time during the day where a transfer back to bed to rest may not be productive due to the rehabilitation schedule (i.e., in between therapy sessions). This time window may present opportunity for increasing physical activity participation independent of traditional therapy times. 

While relatively inactive, the participant was rarely alone. EA was interacting with at least one other individual for 67% of the observations. Of this time, 55.5% of the observations recorded interactions with the therapy staff, 34.4% with family members and 10.1% with nontherapist/nonprofessional hospital staff. Two or more persons were present with the subject during 25% of the person-present observations. 

## 3. Discussion

There are two issues facing optimizing of dosage of rehabilitation therapies and nontherapeutic activity: the dosage necessary to improve the cardiovascular conditioning of the patient to increase independent engagement in daily living skills and the dosage necessary to improve the functional mobility of the patient. The activity pattern for EA suggests that there is considerable “down time” during the rehabilitation day. Some of that down time provides needed rest between therapy sessions. However some time, especially that associated with the “seated but resting” category, may reflect a window that could be capitalized on to increase activity, especially that activity which could increase cardiovascular endurance. Independent seated physical activity (such as getting one's-self to therapies using independent wheelchair mobility, or seated low resistance, high repetition strength training) may be able to be increased in a way that does not require additional staff supervision or increase risk of injury due to falls. 

Although EA receives rehabilitation services consistent with reported norms, it appears that the bulk of the rehabilitation delivered is provided at a relatively low intensity with an emphasis on seated activities. This is a bit surprising given EA's discharge goal of “some household ambulation.” Other studies have reported more inactivity in the stroke recovery process, however, the intensity of services provided seem to be consistent with other reports [6, 7]. 

The examination of EA's activity intensity profile provides a window into the types of interventions delivered, and secondarily into the dosage of interventions targeted specifically to practice of ambulation. The relatively low-activity intensity suggests few upright-, gait-, or pregait-related activities were delivered. Although one explanation for the low physical activity intensity in this case may be attributed to EA's risk of injury with unsupervised upright activity, the activity profile suggests that therapeutic activities may not be challenging the ambulation goal. It is possible that the activity pattern reflects the prioritization of safety over upright mobility. As the therapists did not identify independent upright ambulation as the primary mobility goal after discharge from the stroke unit, a focus on seated mobility may have dominated the rehabilitation sessions. While independent seated mobility may be a very meaningful outcome for assuring safety at discharge, if this goal limits practice of upright ambulation (and thus discharge outcome) perhaps these strategies should be reconsidered. 

Identifying ideal intensity for rehabilitation is a difficult challenge for therapists designing a comprehensive rehabilitation program for a person in the early stages of stroke recovery. Dosage and intensity are informed by many factors; some specific to the individual, some based on the experience of the therapist and team, and some based on resources and externally imposed regulations. In the absence of clear guidelines informing ideal intensity, however, the rehabilitation team should explore whether activity intensity in and outside of formalized rehabilitation sessions is adequate to advance behavioral change desired. 

This case study begins to ask the question as to whether intensity of physical activity is adequate to sustain the long-term behavioral change and cardiovascular conditioning needed in stroke recovery. This particular case begs some interesting questions. EA spent approximately 13 percent of the rehabilitation day either sleeping or resting. While this number is not as high as reported in other studies, perhaps this down time needs further description to optimize outcomes. Additionally, absent more time to utilize, perhaps an increase physical activity of individual therapy sessions could be examined. A comprehensive assessment of rehabilitation from admission to the rehabilitation unit through discharge from rehabilitation should be considered to adequately assess the intensity of dosage of rehabilitation throughout the entire course of formalized stroke recovery. 

This case also demonstrates some limitations to the understanding of stroke recovery. Although the impact of low intensity functional training in the post-acute window of stroke recovery appears to support improvement in functional performance without clear change in underlying cardiorespiratory fitness [14, 15], whether this effect holds in the acute phases of stroke recovery is unknown. By contrast, other authors seem to support an exercise dosage of moderate intensity and short duration (30 minutes) optimize coronary risk reduction for people in the post-acute phase of stroke recovery [16]. A “best” dosage may be dependent upon phase of stroke recovery and individual goals (enhanced function or enhanced fitness and reduced stroke risk). 

Stroke rehabilitation is complex. Interventions promoting functional recovery need to be balanced with interventions designed to assure safety of the stroke survivor and primary caregiver, and interventions that optimize reduction of stroke. What may be best in the acute phases of stroke recovery may not be best in subacute phases of stroke recovery. These changing and sometimes competing needs are delivered in an environment defined by shorter lengths of stay and increased responsibility on the family to provide care. The acute recovery window needs further study to support the rehabilitation team in providing optimized care in a changing resource environment.

## 4. Conclusions

In the United States, inpatient rehabilitation after stroke is characterized by a short, intense bout of therapy provided with the goal of optimizing probability of discharge to home. In the case observed, the physical intensity of the rehabilitation seems relatively low compared to the mobility and self-care expectations of the patient upon return home. The relative inactivity raises questions about adequacy of activity dosage necessary to meet rehabilitation goals.

## Figures and Tables

**Table 1:** Percent time in physical activity during rehabilitation day^a^.

Description of activity	Percent time in activity	Activity code
Sitting supported engaged in cognitive activity	35.92	1
Sitting supported resting	19.42	1
Sitting supported using unaffected UE in therapeutic activity	18.45	2
Sleeping	10.68	0
Sitting unsupported, no activity	3.88	3
Sitting supported engaged in UE activity with affected extremity	2.91	2
Not observed	2.91	n/a
Walking or walking activity	1.94	4
Resting, laying in bed, but not sleeping	1.94	0
Independent seated mobility	.97	4
Sitting in bed in ADL activity	0.97	2

^a^Observations occurred between 7:30 AM and 5:00 PM. Activities from the appendix that were not witnessed are not included in this table.

**Table 2:** 

Observed activity	Activity class (Intensity)	Category	People present (y/n)—classification^a^
No physical activity/resting	No activity	0	
No physical activity/resting but not sleeping (in bed)	No activity	0	
Supported in bed, engaged in therapeutic cognitive activity	Therapeutic activity	1	
Talking/reading/watching television while in bed supported	Nontherapeutic activity	1	
Eating with unaffected hand while in bed supported	Nontherapeutic activity	1	
Sitting in bed supported, ADL activity	Therapeutic activity, low intensity	2	
Sitting out of bed, supported, eating or exercising with the unaffected UE	Therapeutic activity, low intensity	2	
Sitting supported out of bed, resting	Therapeutic activity, low intensity	1	
Sitting supported out of bed, talking/reading/watching or engaged in other cognitive activity	Therapeutic activity, low intensity	2	
Sitting supported out of bed engaged in UE activity with the affected extremity	Therapeutic activity, moderate intensity	3	
Sitting unsupported	Therapeutic activity, moderate intensity	3	
Mobility training exercise (bed mobility, transfer training)	Therapeutic activity, moderate intensity	3	
Mat activities (4 point, kneeling, floor transfers or other core training exercises)	Therapeutic activity, high intensity	4	
Standing activities	Therapeutic activity, high intensity	4	
Walking or walking activities	Therapeutic activity, high intensity	4	
Independent seated mobility (e.g., wheelchair propulsion)	Therapeutic activity, high intensity	4	
Aquatic therapy	Therapeutic activity, high intensity	4	

^a^Classification of persons present; 1: Family member, 2: Nurse, physician, or nontherapist member of the health care team, 3: hospital staff (transporter, aid, interpreter), 4: therapist (physical therapist, occupational therapist, speech pathologist), 5: Physical Therapist Aid or Student Physical Therapist, 6: Psychologist, 7: Certified Nursing Assistant.

## Appendix

Physical activity, intensity, categorization, and social interaction are shown in Table 2.
